# Comment on Giraldo-Ocampo et al. B Cell Subsets in Colombian Adults with Predominantly Antibody Deficiencies, Bronchiectasis or Recurrent Pneumonia. *Adv. Respir. Med.* 2022, *90*, 254–266

**DOI:** 10.3390/arm93030015

**Published:** 2025-06-09

**Authors:** Behnam Shafaei

**Affiliations:** Children Growth Disorder Research Center, Shahid Sadoughi University of Medical Sciences, Yazd 8916978477, Iran; behnamshafaei99@gmail.com

I am writing regarding the article titled “B Cell Subsets in Colombian Adults with Predominantly Antibody Deficiencies, Bronchiectasis or Recurrent Pneumonia” [1] by Sebastian Giraldo-Ocampo, Anilza Bonelo and Andres F. Zea-Vera, published in *Advances in Respiratory Medicine*, 2022.

Common variable immunodeficiency (CVID) refers to a heterogeneous group of primary immune deficiency diseases defined by decreased serum levels of Immunoglobulins (IgG, IgA, IgM), with impaired antibody production [2]. B-cells with CD21low expression are more expanded in these patients compared to healthy individuals [3].

In the fourth part of the Results Section of the article in question, “Alteration of B Cell Subsets Frequencies and Classification of PAD Participants”, it is stated that five CVID patients had more than one percent B-cells, and three out of five had an increase in CD21low B-cells. However, as shown in Table 2 of the paper [1], in which patients are categorized by EUROclass [4], only two of these five patients were CD21lo, and three were CD21norm.

This controversy raises questions about the true count of patients with CD21lo and would affect future studies on CD21lo expansion in patients.

I commend the authors for their decision to clarify the data and report accurate data for the benefit of readers and researchers.
